# Primary care providers’ experiences caring for complex patients in primary care: a qualitative study

**DOI:** 10.1186/s12875-016-0433-z

**Published:** 2016-03-22

**Authors:** Danielle F. Loeb, Elizabeth A. Bayliss, Carey Candrian, Frank V. deGruy, Ingrid A. Binswanger

**Affiliations:** Department of Medicine, Division of General Internal Medicine, University of Colorado School of Medicine, Aurora, CO 80045 USA; Kaiser Colorado, Kaiser Permanente Institute for Health Research, Denver, USA; Department of Family Medicine, University of Colorado School of Medicine, Aurora, Colorado USA

**Keywords:** Chronic disease, Doctor-patient relationship, Medical comorbidity, Primary care, Work related stress, Medical home/patient-centered medical home

## Abstract

**Background:**

Complex patients are increasingly common in primary care and often have poor clinical outcomes. Healthcare system barriers to effective care for complex patients have been previously described, but less is known about the potential impact and meaning of caring for complex patients on a daily basis for primary care providers (PCPs). Our objective was to describe PCPs’ experiences providing care for complex patients, including their experiences of health system barriers and facilitators and their strategies to enhance provision of effective care.

**Methods:**

Using a general inductive approach, our qualitative research study was guided by an interpretive epistemology, or way of knowing. Our method for understanding included semi-structured in-depth interviews with internal medicine PCPs from two university-based and three community health clinics. We developed an interview guide, which included questions on PCPs’ experiences, perceived system barriers and facilitators, and strategies to improve their ability to effectively treat complex patients. To focus interviews on real cases, providers were asked to bring de-identified clinical notes from patients they considered complex to the interview. Interview transcripts were coded and analyzed to develop categories from the raw data, which were then conceptualized into broad themes after team-based discussion.

**Results:**

PCPs (*N* = 15) described complex patients with multidimensional needs, such as socio-economic, medical, and mental health. A vision of optimal care emerged from the data, which included coordinating care, preventing hospitalizations, and developing patient trust. PCPs relied on professional values and individual care strategies to overcome local and system barriers. Team based approaches were endorsed to improve the management of complex patients.

**Conclusions:**

Given the barriers to effective care described by PCPs, individual PCP efforts alone are unlikely to meet the needs of complex patients. To fulfill PCP’s expressed concepts of optimal care, implementation of effective systemic approaches should be considered.

**Electronic supplementary material:**

The online version of this article (doi:10.1186/s12875-016-0433-z) contains supplementary material, which is available to authorized users.

## Background

Complex patients, defined by the Agency for Healthcare and Quality (AHRQ) as persons with two or more chronic conditions where each condition may influence the care of the other condition, are commonly cared for in primary care [1]. Complex patients with multiple chronic conditions have increased medical costs, a higher number of preventable complications, higher rates of avoidable hospitalizations, and decreased quality of life [2, 3]. In 2006, over 25 % of adult patients and 75 % of those over 65 reported having more than one chronic condition [4]. One study of 148 primary care practices found that 45 % of adult patients had two or more chronic conditions [5]. Family physicians in Wisconsin reported addressing more than four problems per visit in over half of the visits for diabetes care [6].

Complex patients’ experience well-described barriers to self-care have been well studied [7, 8]. Less is know from the provider perspective. Physicians and pharmacists have highlighted a lack of time, poor communication with specialists, and fragmented care as barriers to effective care [9]. In studies focused on clinical decision-making for patients with multiple chronic conditions, a lack of time and adequate reimbursement emerged as barriers to clinical decision-making [10, 11].

Although barriers to medication prescribing and decision-making for primary care physicians (PCPs) caring for complex patients have been previously described, less is known about the potential impact and meaning for PCPs of caring for these patients on a daily basis. Developing a greater understanding of PCP experiences caring for complex patients is important because providing such care in the context of scarce time and resources may be associated with physician burnout [12, 13]. Burnout in PCPs has been associated with a higher likelihood of leaving practice [14]. Among New York PCPs, participants described significant time pressures, chaotic work pace, and low level of control over their work [15]. In the United Kingdom, General Practitioners described tension between addressing their patients’ agendas and meeting quality measures, which increased when treating patients with multiple chronic conditions [10]. In the 2015 Commonwealth Fund International Health Policy Survey of Primary Care Physicians, only 16 % of US physicians felt that the healthcare system worked well, 33 % felt the quality of care patient received in the healthcare system had gotten worse in the last 3 years, 43 % stated their job was very or extremely stressful, and 34 % were somewhat or very dissatisfied practicing medicine [16]. Thus, the increasing patient complexity in the setting of limited time and clinical support may represent an important source of career dissatisfaction and burnout for PCPs [17].

Little is known about the strategies PCPs employ when faced with challenges providing care for complex patients. In order to better understand the day-to-day challenges of PCPs, the strategies they use to meet these challenges, and how to better support PCPs, we sought to characterize PCPs experiences with complex patients. Through open-ended interviews we explored their perceived barriers and facilitators to effective care and their strategies to address barriers.

## Methods

### Study design, setting, population and recruitment

Using a general inductive approach, our qualitative research study was guided by an interpretive epistemology. Because interpretive traditions take human interpretation as a starting point for developing knowledge about the social world, discourse, and its resulting accomplishments, was central to this study to understand participants’ values, beliefs and motivations. Therefore, we conducted a qualitative study of PCPs using previously described open-ended in-depth interviews [18, 19]. Briefly, we recruited 15 internists from two university-based clinics (UCs) and three safety net community health clinics (CHCs) affiliated with the University of Colorado School of Medicine. We invited participation by sending email notices to all physicians in the practices (34 and 28 physicians from the UCs and the CHCs, respectively). We used systematic non-probabilistic sampling [20] to achieve an even distribution of gender, years in practice, and practice location. In line with interpretative and participatory forms of research, participants were viewed as active agents participating in the actual co-construction of meaning, rather than mere subjects representing and reproducing meaning. This distinction mattered for how we classified, organized, and interpreted the data focusing on meaning and intentionality over and above casual explanations. We came to agreement about what is real intersubjectivly, which informed our ability to generate solutions, grounded in lived experience, for handling complex patients. We completed the interview process after 15 interviews with 8 UC and 7 CHC participants after reaching thematic saturation.

### Instrument and interview procedures

**Fig. 1:**
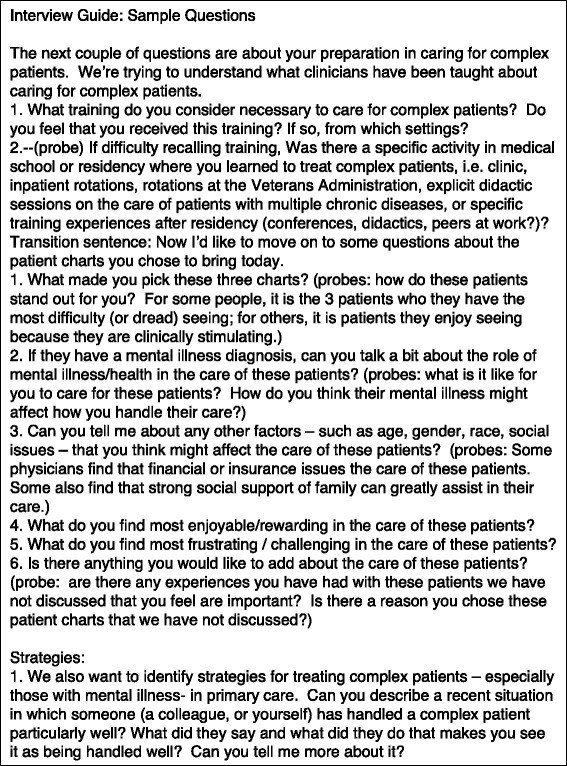
Interview Guide: Sample Questions

**Fig. 2:**
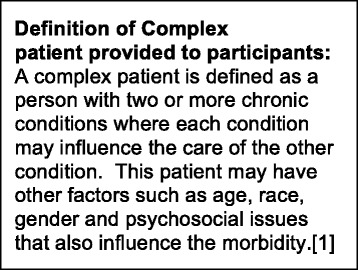
Definition of complex patient provided to participants [1]

The interview instrument was developed by the Primary Investigator (D.F.L) and revised iteratively through input from the full research team and other qualitative and health services researchers. It was then refined after three pilot interviews. The interview guide was designed to elicit PCPs’ views of patient factors, physician competence, and healthcare system issues associated with caring for complex patients (Fig. 1). The guide also included questions about the role of mental illness in patient complexity. Because we were specifically interested in interpreting facilitators and barriers to care, our analysis sought to identify PCP strategies employed to improve care. We started the interviews by providing participants with a definition of complexity (Fig. 2), thereby allowing us to define a common focus of study (i.e., patient complexity) while allowing underlying experiences or processes related to complexity to also emerge from the data.

**Fig. 3:**
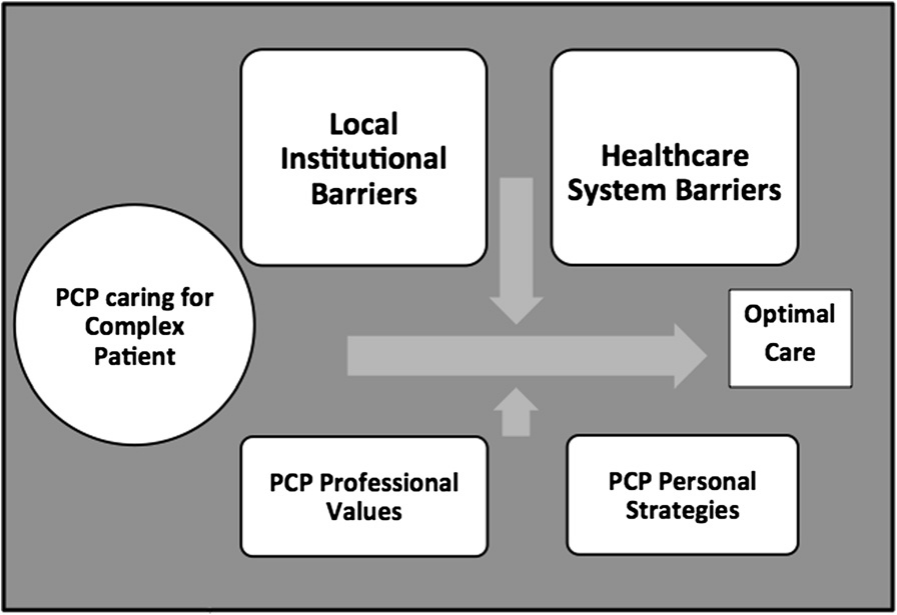
Schematic Illustration of Primary Care Providers (PCPs) Caring for complex patients in context of local and healthcare system barriers with inadequate resources

Participants were asked to bring de-identified clinical notes from three patients who met the definition to the interview to keep the discussion focused on concrete cases seen in clinical practice rather than broad generalization. Participants were also asked to complete a brief demographic survey.

The Primary Investigator (D.F.L.), a PCP, and another investigator (C.C.), conducted the interviews from July through December 2010. The one-on-one interview format was selected to allow deep exploration of individual clinician experiences and reactions to these experiences in a confidential setting [21]. Participants were not compensated. Interviews were conducted in a private space, lasted approximately 60 min, were digitally recorded, uploaded to a secure drive, and professionally transcribed.

### Analysis

Interview transcripts and a demographic survey were our primary data sources. Transcript files were entered into qualitative data analysis software (ATLAS.ti Scientific Software Development GmbH, Berlin, Germany). We used an interpretive and participatory approach to explore patterns and themes [22, 23]. Members of the team included health services researchers, including: two general internists (D.F.L and I.A.B) and two family medicine physicians (E.A.B. and F.V.D.) and a Doctor of Health Communication (C.C.). The PI (D.F.L.) and another team-member (C.C.) conducted the interviews. Recognizing our unique positions and our own interpretive frameworks, the analysis was guided by qualitative research ethics that allowed us to interpret interviews not simply as representational of all PCPs. Rather, since the research team consisted of PCPs and health services researchers, our interpretation involved a generative discussion of meaning with the potential to better understand our shared situation around complex patients [24].

D.F.L. performed initial coding using an inductive coding approach [22]. Codes were initially broadly categorized according to the domains of patient care explored in the interviews [25]. Then, two other team members (I.A.B. and E.A.B.) independently coded two of the interviews and worked with the primary coder to resolve differences and create the final codebook. Other team members (C.C. and F.V.D.) reviewed a subset of transcripts. The full team met to discuss emerging themes and discrepancies. We coded data for manifest content meaning (surface content, e.g., PCPs shared that helping complex patients avoid hospitalizations was rewarding for them) and latent content meaning (underlying meaning, e.g., grouping the activity of helping patients avoid hospitalization into the category of optimal care) [26].

COREG checklist can be viewed online (Additional file 1). This study was approved by the Colorado Multiple Institutional Review Board. All participants signed written consent to participate in the study.

## Results

Participant characteristics (*N* = 15) are described in Table 1.

**Table 1:** Participant Characteristics

Participant characteristics (*n* = 15)	
Age in y, mean (range)	38 (29–52)
Female, *n* (%)	9 (60)
Race/ethnicity, *n* (%)	
White Non-Hispanic, *n* (%)	12 (80)
Asian, *n* (%)	2 (13)
White Hispanic, *n* (%)	1 (7)
Site of practice	
Community Health v. University, *n* (%)	7 (47)
Time since residency completion in y, mean (range)	8 (<1–24)
Time in primary care practice in y, mean (range)	7 (<1–24)

### Overview of PCP experiences

PCPs described complex patients as those with multidimensional needs, such as socio-economic, medical, and mental health. A vision of optimal care for complex patients emerged from the data, but PCPs also encountered significant local and larger healthcare system level barriers to providing such care. To overcome these barriers, PCPs depended on professional values and individual care strategies. We developed a schematic illustration to illustrate this problem of inadequate PCP resources to meet the challenge of caring for complex patients (Fig. 3).

### Complex patients

Participants described complex patients as those with medical, mental health, and social needs. One CHC participant described a typical highly complex patient:

*[This 52-year-old patient] has diabetes, hypertension, gout, hyperlipidemia, peptic ulcer disease, asthma. His med list is at least two pages long. He was homeless and had depression and schizoaffective [disorder]… He had only been out of jail for a couple weeks. He had no insurance or anything…. He wasn’t eating regularly, so he was afraid to take his insulin…*

### Optimal care

Participants described the needed care for complex patients and PCPs’ ideal role in this care, which we termed “optimal care”. We defined optimal care as the activities they described performing in their role as PCPs that were most helpful to their patients and rewarding for them. Coordinating care, preventing hospitalizations, and building patient trust were identified as key elements of optimal care.

### Coordinating care

For most participants, managing discussions among specialists and helping patients to make informed decisions was one of their most important roles. One CHC participant discussed the importance of the PCP leading the healthcare team:

*I also think that for some things you just need to have someone who is in charge. For the complicated patients who haven’t done well, nobody has said, ‘I'm in charge. I'm taking care of you’. You know, ‘I’m going to be the captain of this ship.’*

One UC participant described coordinating care for her complex patients as both necessary and fulfilling.

*Every time I see him I’ve ended up… contacting 6 or 7 other providers. We’ve all decided that’s what it is going to take to make sure that this individual gets what he needs… I feel like I do have a role and I really feel like I’m fulfilling my role as a coordinator for him…and I see it helping him become more confident in his decisions and about his ability to take care of himself.*

### Preventing hospitalizations

Many participants were highly satisfied helping their patients avoid hospitalizations by care coordination, frequent follow-up visits, and careful attention to their medical regimens. One CHC participant explained, “Being outpatient, many things can fall through. When you can make a smooth transition for a patient without them ending up in the hospital… that is a success story.”

### Building trust

PCPs discussed developing a trusting relationship with their patients as therapeutic for patients and as fundamental to their ability to provide optimal care. One UC physician reported:

*[I]t’s almost like we’re attached to each other. [Complex patients] always know that even if all the other doctors [specialists] they are seeing aren’t listening, they can come and have a central place where I can help coordinate. So I feel like they trust me, which is probably the most rewarding aspect of their care.*

### Healthcare system factors

PCPs described healthcare system factors that interfered with their ability to provide optimal care, including payment systems, patient insurance issues, poor access to mental healthcare, and the fragmentation of care.

### Payment systems

Some UC participants expressed the belief that the prevalent fee-for-service payment system undermined effective patient care, because payment was focused on discrete services rather than coordination of care. One UC PCP described large amounts of uncompensated time coordinating care:

*A patient I take care of who has multiple sclerosis comes in once a year…otherwise it’s all done by phone or communication with her visiting nurse. Over the course of a year I might spend 15 or 20 h on her care. I get paid for the 40 min that she spends in my office once a year.*

### Uninsured or underinsured patients

Both UC and CHC participants cited lack of insurance or underinsurance as a barrier to optimal care for complex patients. For example, on UC PCP explained:

*[H]e has insurance, but with all of his 20 different medications, it costs him over $200 a month for his prescriptions. So he [rations]…he doesn’t take all of his meds… This is a guy who works 40 h a week, who has insurance, but still can’t afford everything.*

### Poor mental health care access

PCPs at all clinics expressed frustration with challenges in accessing mental health care. One UC participant discussed the lack of resources for patients with suicidal ideation:

*I think the access to mental health assistance is so poor… You know, somebody comes in and says they are going to hurt themselves, our only option is to send them to the emergency room… Often times you can’t get them to be seen anywhere.*

### Fragmentation of care

PCPs described barriers that were consistent with the concept of care fragmentation, defined in the literature as “having multiple decision makers make a set of health care decisions that would be made better through unified decision making” [27], interfered with patient care. This issue was particularly salient for patients with mental illness. One UC PCP expressed frustration over the challenges he found communicating with psychiatrists outside of his system. Referring to patients seen at a local behavioral health organization: “They don’t use the same record system. So, I can’t see what the psychiatrist is doing if they [my patients] do manage to land in a psychiatry office. It is hard to call and speak with them.”

### Local system factors

Within local healthcare systems, participants struggled with insufficient clinical support, challenges communicating with specialists, and productivity demands.

### Insufficient clinical support

Providers reported lack of case management and social work support in their local clinics as important barriers to optimal care. Social workers were identified as an important needed resource to improve care for complex patients. One PCP from a UC stated:

*We essentially don’t have the social worker.... I mean getting people to appointments and making sure they have [transportation service] or does someone need to be checked up on. We don’t have help with any of that, and, to be honest, a lot of it just goes to the wayside because we don’t have help…*

While participants in the CHCs did have a social worker, they did not have case management or mental health services. With case management support PCPs believed they could more effectively use their time.

### Communication with specialists

While communication with specialists was fundamental to PCP’s ability to provide comprehensive care to complex patients, many participants described this communication as time consuming and, at times, impossible. One UC PCP explained, “The reality is, you can try to page the doctor [specialist]… but you end up leaving a message and then they may or may not get back to you.”

### Productivity demands

PCPs at both UCs and CHCs expressed frustration over productivity demands. CHC PCPs focused on lack of flexibility with visit length.

*There is no ability to have more time with individual patients. If you are a provider who has a lot of complicated patients, that doesn’t affect [i.e., lower] your productivity expectations or [lower] your panel size expectations.*

UC PCPs focused on pressures around billing and compensation unrelated to patient care:

*[T]he things that really matter have almost nothing to do with how we’re paid. In order to take care of these people, we have to have the background knowledge of their life experiences and their multiple diseases and their multiple medicines. Their spouse died of cancer and they’ve had problems with depression and a life long history of alcohol… What we get paid for is…[how] many body parts we examine and…[how] many different things that we’ve asked them…*

### PCP professional values

When discussing successful approaches to managing patient complexity, participants emphasized the importance of values of professionalism and self-reflection. They relied on these professional values to provide optimal care in the face of significant system barriers.

### Professionalism

The theme of professionalism emerged, which was described as rendering appropriate care despite barriers. PCPs ensured patients received the care they needed, despite inadequate resources, by spending extra time caring for complex patients. Personal sacrifice and the placement of patient needs over their own personal needs were noted as integral to the provision of effective care. For example, one UC participant stated: “So really I think, if you approach it with an attitude of service that you are a servant to these people and you really have to kind of efface your own needs…”

Another UC participant discussed the importance of following complex patients closely regardless of whether those activities are compensated:

*Like sometimes it is like staying late at night to call a patient when they are in the hospital somewhere to make sure they are going to make an appointment to come back in and be seen. Or you know, you make a referral and hey, I noticed you didn’t go to your referral. What’s going on? Why didn’t you make it? … It takes time and we don’t get paid for any of that. Nobody cares whether or not you stay late to do that.*

A CHC participant expressed a similar idea, “And the professional piece, realizing that sometimes I do need to work through lunch with the dementia patient where the family is fighting.” However, that PCP continued to note the possible consequence of self-sacrifice, “I mean the flip side of that is I’m so frustrated with our system that I don’t do a lot of clinic.”

### Self-reflection

PCPs emphasized the importance of self-reflection when treating complex patients, especially those with challenging communication styles. A UC PCP explained, “I guess I try to be careful. When something about a patient makes you unhappy or uncomfortable, some of it has to be about you, you know? And so I think just recognizing maybe it’s not all them.”

### PCP personal strategies

PCPs described the following strategies for taking care of complex patients: getting to know their patients, frequent visits, prioritizing issues, and setting limits.

### Getting to know patients

The most common strategy PCPs expressed was the importance of getting to know their patients. Once PCPs understood patients’ complex milieu of medical, mental health, and social issues, they were better able to prioritize issues and help their patients make healthcare decisions in alignment with their goals. A CHC PCP described, “[it’s] seeing where they are and where they are willing to go and to try to meet them part way.” The PCP offered the following example:

*The one gentleman who has had the significant alcohol problem … it wasn’t that he admitted he was going to quit drinking, because he is just nowhere near that right now, but he was willing to admit the amount he is drinking and the amount he is eating, is really not good for his overall health. And he knows the alcohol is not good for his overall health. But he doesn’t really want to starve to death. And that is sort of what he’s doing right now. And so he is willing, maybe, to cut back on the alcohol and eat a little bit more. I mean it’s not really that much of an accomplishment, but I think it’s a start …*

Another CHC PCP explained the personal reward of getting to know patients well:

*I think in talking to people and realizing what else is going on with them, and that is driving some of their difficult or not adherent behavior, or other things, I find that to be rewarding.*

### Frequent visits

PCPs scheduled frequent visits with their complex patients to avoid crises, lapses in the medication treatment, and hospitalizations.

### Prioritizing issues

One of the strategies emphasized was prioritizing issues during patient visits. A CHC PCP suggested, “Try to focus on whatever issues are most going to affect the patient’s health in terms of their quality of life and their ability to function.”

### Setting limits

Setting limits with individual patients emerged as a theme. Setting limits was particularly important in caring for patients with chronic pain requesting opioids and with patients with challenging communication styles. On CHC PCP explained their approach, “I go in there, and I’m honest: This is what I’m going to do; this is what I’m not going to do; and, then, that’s the way I follow through.”

### Systemic changes

Participants described systemic changes to their practice that would improve the care of complex patients, such as additional assistance from team members. One UC participant expressed the need for team-based approaches: “[I]t really needs to be more than just the physician… I mean especially with the complex patients.” The UC clinics were starting to transition to a Patient Centered Medical Home (PCMH) model. The same provider described positive early experiences with this model.

*We have switched to a Medical Home team orientation over the last year and it’s been extremely rewarding… for a medically complex patient it helps… to have other staff help proactively monitor people’s complex conditions.*

While participants had minimal experiences with team-based models of care, some perceived teams as strategies to meet the challenges involved in caring for complex patients.

## Discussion

In this study, we used in-depth semi-structured interviews to understand PCP experiences treating complex patients in primary care settings. PCPs described: 1) strong desires to provide optimal care for complex patients with multidimensional needs, 2) significant barriers to optimal care delivery at a local and system level, and 3) reliance on professional values and individual care strategies to overcome these barriers.

PCPs described optimal care for their complex patients in terms of actions they could take that would help their patients’ overall health and quality of life. Specifically, they focused on coordinating care among specialists, preventing hospitalizations, and building patient trust. This description of optimal care aligns with recommendations for a shift from problem-oriented care to goal-oriented care [28, 29]. Goal-oriented care focuses on patient-defined goals rather than disease-specific guidelines. This shift has been supported by research that highlights potential negative consequences of following guideline-concordant care for multiple different concomitant diseases in the same patient [30].

Within their local institutions, PCPs reported that insufficient clinical support, challenges communicating with specialists, and productivity pressures interfered with their care of complex patients. These findings are consistent with past qualitative research on prescribing and decision-making for complex patients [9–11]. Further, the time pressures and lack of clinical support raise a risk of burnout in these providers. In the Minimizing Error, Maximizing Outcome (MEMO) study, adverse workflow (time pressure and chaotic environments), low work control, and unfavorable organizational culture were strongly associated with low physician satisfaction, high stress, burnout, and intent to leave [15].

PCPs relied on professional values and personal strategies to navigate local institutional and healthcare system barriers to provide optimal care to their complex patients. This emphasis on professionalism, specifically self-sacrifice, and self-reflection by PCPs in this study is consistent with core values of professionalism articulated by physician professional organizations. Professionalism has been identified as one of six Core Competencies for internal medicine residents [31]. American and European Internal Medicine boards and societies [32] have identified 1) the primacy of patient welfare; 2) patient autonomy; and 3) social justice as three principles of professionalism. While reliance on professionalism and self-sacrifice is consistent with core competencies, it may not overcome the system barriers preventing optimal care. Further, since professionalism often was described as working longer hours and sacrificing PCP’s personal needs, these strategies may contribute to PCP burnout.

Participants endorsed changes consistent with team-based care models to help them effectively manage complex patients. Team-based interventions [33, 34] based on the Chronic Care Model [35] have been developed for managing high-risk [36] patients with chronic diseases in the primary care setting. Transitioning to team-based models may address some of the barriers to optimal care identified by PCPs in our study. Among VA primary care personnel, those working in clinics that were appropriately staffed, emphasized participatory decision making, and had an increased proportion of time team members spend working to the top of their competency level reported lower rates of burnout [37]. Working in a tight team structure and greater perceptions of team culture have been associated with less clinician exhaustion [38].

## Conclusion

Readers should generalize these findings with caution. First, all participants were affiliated with a single university in the Rocky Mountain region, but worked diverse settings, including an academic medical center and urban, community health centers. These clinical sites were chosen because of their concentration of complex patient populations. Second, the providers in this study were relatively young and most had been in practice for less than 10 years. Physicians in practice for longer may develop greater skills for managing complex patients. Third, four of the five researchers who conducted this study were PCPs. Thus, the analysis was likely influenced by their own professional roles, which may have informed a rich understanding and generative discussion of meaning regarding the care of complex patients [24]. Lastly, we used the term “complex patients” in materials with participants because it is commonly used in the United States when referring to patients with multiple chronic conditions. However, we have some concern that this term can have a pejorative connotation. AHRQ has more recently been using the term “patients with complex care needs”. Other commonly used terms include multimorbidity and multiple chronic conditions.

Our study identified core struggles in the daily practice of managing complex patients. PCP reliance on professional values and individual strategies to manage systemic challenges may not necessarily produce optimal care and may contribute to PCP burnout. It remains to be shown whether these barriers to optimal care can be reduced with new models of care.

## Additional file

Additional file 1. COREQ (COnsolidated criteria for REporting Qualitative research) Checklist. (PDF 280 kb)
